# How Can We Safeguard Our Teeth?

**Published:** 1923-10

**Authors:** 


### HOW CAN WE BEST SAFEGUARD OUR TEETH?

There is probably no other problem in the field of preventive medicine deserving of more careful study and serious consideration than that of the care of the teeth. This problem, unfortunately, has afforded many an enthusiast an excellent opportunity to permit his enthusiasm to warp his judgment. Not long since, one of these, occupying a prominent position in the United States, stated that in a certain city where the principles of oral hygiene have been carefully tried out, diphtheria, measles and scarlet fever have been reduced more than one-half as a direct result. We know, as a matter of fact, that neither he nor anyone else could possibly justify any such claim if the records were followed up year by year for five or ten years.

The same writer makes most extravagant claims about emptying asylums by cleaning up the mouths of the inmates, stating that by such measures even as high as seventy per cent. in one asylum have been discharged and that in addition to this there has been a wonderful reduction in the number of cases of mentally sub-normal children.

Such statements are most unfortunate, inasmuch as there is ample, indisputable evidence of the important role played by dental caries and by the general disregard of the care of the teeth in the health of the individual.

If it were for no other reason, the preservation of the teeth for the purpose of mastication would more than justify all the efforts that we could put forth. In addition to this, there is an extremely important role that teeth play as a source of focal infection from pyorrhea and from abscess formation at the roots of the pulpless teeth. Pulpless teeth that have been veneered with gold crowns and bridges have called down the anathemas of the medical profession and of modern students of dentistry. Teeth, tonsils and the intestinal canal form a triple alliance of focal infection.

In Toronto, as in all large cities, where surveys have been made of the schools, it has been found that from eighty-five to ninety-five per cent. of the children entering school have one or more defective teeth, or from one to ten cavities in every one of the children's mouths. However, after preventive measures have been rigidly carried out after entering school and an educational campaign carried on among the children previous to entering school, we are finding that the percentage of children with defective teeth is becoming lower and lower year by year.

### DIET VERSUS TOOTH BRUSH

When Carlyle said that only one person out of five thousand thinks, he might have added that many of those who do think, do so only on the instalment plan, or that their intellects seem to become short-circuited after a brief activity along certain lines.

For generations, the medical profession has known that defective or arrested bone development in the case of rickets was due to some deficiency in the diet, a lack of calcium, carbon or phosphorus, and that the continued use of cod liver oil, milk, cream, eggs, was promptly followed by rapid improvement. However, it is only in recent years that we have learned that cod liver oil and cream are two of the most valuable sources of anti-rachitic vitamins. Children suffering from rickets invariably have defective teeth. Why did we not realize sooner defective teeth resulted from defective diet?

We find in all museums, skulls of people of advanced years, the jaws of which are studded with well developed teeth in good condition. When those who have been making observations along these lines examined these skulls, did it never occur to them that the tooth brush was unknown to these early peoples as were also the tooth powders and tooth washes? They had, however, the most valuable of all tooth washes, a free flow of saliva. They had sound teeth because they embraced in their diet, the essentials for the development both of bone and that hardest, most resistant of body structures, the enamel of the teeth.

Professor McCollum of Hopkins draws attention to the collections of skulls of Indians from the Aleutian Islands, Peru, Honduras and the South Sea Islands in the National Museum at Washington, who lived between two and three hundred years ago. Among several hundreds of these, only one tooth showed dental caries. He states, in tracing the history of these people and their methods of living, that their diet was excellent from the standpoint of chemical completeness. He further emphasizes the fact that the conditions found in these people, hold good in all pastoral people where dairying is the leading industry, and ample amounts of milk and dairy products are available.

It is not necessary, however, for us to go back to the crude, coarse food of our forefathers, in order to develop proper jaws and teeth. It is essential, that we see to it, that our food contains an ample supply of material from which jaws and teeth are made. Furthermore, we are not aware that Nature discriminates in its distribution of gifts, but sees to it that the back is fitted for the burden, and consequently on account of the extra strain brought to bear upon the teeth at all times, and their exposure to deteriorating influences, such as extremes of heat and cold and bacterial infection, they are provided with a covering of enamel. The consistency or quality of this enamel is in direct ratio to the material supplied in the diet of the individual.

Much has been said and written recently on the influence of the sun's rays and the ultra-violet rays in the prevention and cure of rickets, used in conjunction with a plentiful supply of milk, cream and butter. It has also been pointed out how much more rapidly these cases of rickets improve in summer. May this not be due in a great measure, to the fact that in summer the dairy cows live largely on grass and green foods of all kinds and consequently their milk is much richer in all of the vitamins, particularly the anti-rachitic vitamin, than it is in the winter when they have not the advantage of green food.

However, it must be apparent that the conditions that influence rickets or that are responsible for rickets, must also play an important role in the definite development of the teeth and consequently in dental decay.

In the light of the knowledge we now possess, as regards the significance of a properly balanced diet, and the role played by the accessory food factors or vitamins, it must be apparent that in our so-called preventive treatment or oral hygiene, and also in our efforts to repair at the earliest moment the damage done, we are simply trying to atone for our failure in prevention. So far as the present generation is concerned, we must continue rigidly carrying out these principles of oral hygiene, but we must bear in mind in the interests of the infant and the very young child and the children yet unborn, and for future generations, that proper nutrition, containing all the accessory food factors, is the foundation upon which preventive dentistry must be constructed. This problem of proper nutrition must begin with the expectant mother and continue through adolescence to maturity.

There have been all sorts of statements made, speculative many of them, regarding the cause of the decay of the teeth. Miller's theory of lactic acid produced by bacterial action on the carbohydrate elements of the good is generally accepted. The lactic acid dissolves the tooth structure and allows the entry of the bacteria which produce decay. It is consequently not an infrequent statement, by those that pin their faith to the tooth brush, that a clean tooth never decays, and that if people continue to neglect these principles of oral hygiene and the early attention to the teeth, in a few generations the race will be toothless. That is probably true if we depend on the present principles of oral hygiene alone. Much can be done by the carrying out of the hygienic precautions in the care of the teeth and the prompt repair of any slight decay. However, we may unhesitatingly state that if we begin with the proper nutrition of the mother, that is, if we see that the expectant mother and the nursing mother has in her food the material out of which perfect teeth and jaws are made, we may expect in a few generations that we will have teeth that will compare favorably with our forefathers and the primitive races.

Defective teeth, adenoids and enlarged tonsils, are all much more prevalent among the undernourished children. Therefore, when we give the attention to the whole problem of nutrition that its importance merits, we will have better teeth, fewer adenoids and enlarged tonsils and a much fitter race.

### WHAT DIET DO WE RECOMMEND

For the expectant mother we only require to bear in mind that the sources of all the vitamins or accessory food factors are in the dairy products and green leaf vegetables, grasses, etc. Of the leafy vegetables, lettuce, spinach and cabbage, for the expectant mother, are the most valuable. There is a liberal amount of two of these vitamins contained in fresh vegetables, but they can not take the place of the green leafy vegetables, some of which should constitute a part of the daily diet of the expectant mother. And in addition to these, with meat, eggs, etc., she should drink at least a quart of milk a day to insure an amply supply of the third vitamin. It is important in this connection to bear in mind that milk contains these vitamins in proportion to the amount of green food that the dairy cow is getting.—*Dominion Dental Journal.*

